# Is *Toxoplasma Gondii* Infection Related to Brain and Behavior Impairments in Humans? Evidence from a Population-Representative Birth Cohort

**DOI:** 10.1371/journal.pone.0148435

**Published:** 2016-02-17

**Authors:** Karen Sugden, Terrie E. Moffitt, Lauriane Pinto, Richie Poulton, Benjamin S. Williams, Avshalom Caspi

**Affiliations:** 1 Department of Psychology & Neuroscience, Duke University, Durham, North Carolina, United States of America; 2 Duke Center for Genomic & Computational Biology, Duke University, Durham, North Carolina, United States of America; 3 Department of Psychiatry and Behavioral Sciences, School of Medicine, Duke University, Durham, North Carolina, United States of America; 4 Social, Genetic, and Developmental Psychiatry Centre, Institute of Psychiatry, King’s College London, London, United Kingdom; 5 Department of Psychology, University of Otago, Dunedin, New Zealand; Albert Einstein College of Medicine, UNITED STATES

## Abstract

**Background:**

*Toxoplasma gondii (T*. *gondii)* is a protozoan parasite present in around a third of the human population. Infected individuals are commonly asymptomatic, though recent reports have suggested that infection might influence aspects of the host’s behavior. In particular, *Toxoplasma* infection has been linked to schizophrenia, suicide attempt, differences in aspects of personality and poorer neurocognitive performance. However, these studies are often conducted in clinical samples or convenience samples.

**Methods/Results:**

In a population-representative birth-cohort of individuals tested for presence of antibodies to *T*. *gondii* (N = 837) we investigated the association between infection and four facets of human behavior: neuropsychiatric disorder (schizophrenia and major depression), poor impulse control (suicidal behavior and criminality), personality, and neurocognitive performance. Suicide attempt was marginally more frequent among individuals with *T*. *gondii* seropositivity (*p* = .06). Seropositive individuals also performed worse on one out of 14 measures of neuropsychological function.

**Conclusion:**

On the whole, there was little evidence that *T*. *gondii* was related to increased risk of psychiatric disorder, poor impulse control, personality aberrations or neurocognitive impairment.

## Introduction

*Toxoplasma gondii (T*. *gondii)* is an obligate protozoan parasite of all warm-blooded mammals including humans, where infection has been linked to schizophrenia, suicide attempt, differences in aspects of personality and poorer neurocognitive performance. In humans, the primary source of infection is through contact with the feces of infected animals, especially domestic cats, the definitive host in which the *T*. *gondii* protozoan completes its life cycle. Alternate sources of infection occur through contact and ingestion of infected meat (especially pork), maternal-fetal transmission and exposure to soil and water contaminated with oocytes. *T*. *gondii* is geographically omnipresent, and it is estimated that age-adjusted country-level seroprevalence ranges from around 3% in South Korea to 76% in Costa Rica [1].

In rare cases, Toxoplasmosis can present with severe pathological symptoms, including retinochoroiditis, myocarditis and meningoencephalitis, potentially leading to death [2]. However, most infected humans are asymptomatic, exhibiting few or no physiological symptoms. Because of this asymptomatic nature, it was long thought that latent *T*. *gondii* infection was of little public health significance except in cases of concurrent immunosuppression, such as HIV infection. However, recent reports have suggested that infection with *T*. *gondii* might have previously unrecognized consequences in humans.

One such consequence concerns host behavioral manipulation. In terms of the relationship between the intermediate and the definitive host, modifying normal interactions between the two would be advantageous to the parasites’ transmission and reproduction. In other words, modification of a rodent’s normal aversive reaction to felines would be advantageous to the parasite, since it would generate a higher chance of it being consumed by the cat, the organism in which the parasite’s life cycle is completed [3]. Indeed, evidence exists that infection of mice by *T*. *gondii* predicts innate loss of fear of cat urine [4] and impaired working memory [5]. These observations have led to the hypothesis that such manipulations might not be unique to rodent hosts. As such, extrapolations of these phenomena to correlates of human behavior are gaining attention.

In line with observed effects of infection on rodents’ behavior, most human research has focused on behavioral domains involving psychiatric illness, impulsivity and aberrant neurocognitive processes. The most heavily researched correlate of *T*. *gondii* infection is schizophrenia. Interestingly, some acute cases of *T*. *gondii* infection result in hallucinations, a key feature of schizophrenia, and reports of inflated numbers of *T*. *gondii* positive individuals in samples of psychiatric inpatients were made as early as the 1950’s [6]. The culmination of these findings is a recent meta-analysis of 38 studies, which suggests that *T*. *gondii* infection increases the odds of developing schizophrenia 2.7 times (OR, 2.71, 95%CI 1.93–3.80) [7]. In addition, links with *T*. *gondii* have also been suggested with major depressive disorder; however, these suggestions have been more hyperbolic. One case report demonstrated alleviation of depressive symptoms upon successful *T*. *gondii* treatment [8], whilst another reported a correlation between cat bites and depression in women [9]. However, association studies of *T*. *gondii* infection with depression have been inconsistent [10, 11].

Poor impulse regulation, including violent and risk-taking behavior, is another potential consequence of infection. Latent *T*. *gondii* infection has been associated with increased human trait aggression in females and increased impulsivity in males [12]. Other studies have reported links between *T*. *gondii* antibody titer and suicide attempt [13–15]. Complementing these individual-level studies, cross-national comparisons have documented that national seroprevalence rates of *T*. *gondii* antibodies titers are positively correlated with higher nation-wide rates of both suicide and homicide [1, 16, 17]. Yet another study has reported higher incidence in seropositivity amongst prison inmates compared to controls [18]. Further reports show that *T*. *gondii* seropositivity is related to both fatal and non-fatal traffic road accidents [19, 20], presumably reflecting poor impulse regulation.

Finally, there is some evidence that neurocognitive and personality differences exist between seropositive and seronegative individuals. In particular, slower reaction times [21, 22] and poor attention [23] appear to be associated with *T*. *gondii* seropositivity, along with lower scores of Novelty Seeking [24].

This study tested the hypothesis that *T*. *gondii* infection is related to impairment in brain and behavior, as measured by a range of phenotypes encompassing neuropsychiatric disorders, poor impulse control, personality and neurocognitive deficits. We tested the hypothesis in a population-representative birth cohort of adults enrolled in the Dunedin Longitudinal Study. We drew on data derived from psychiatric interviews, neuropsychological testing, and a search of administrative records to conduct, to our knowledge, the most comprehensive evaluation to date of the link between *T*. *Gondii* infection and the full panoply of presumptive impairments.

## Materials and Methods

### Participants

Participants are members of the Dunedin Multidisciplinary Health and Development Study, a longitudinal investigation of health and behavior in a population-representative birth cohort [25]. Study members (N = 1,037; 91% of eligible births; 52% male, 48% female) were all individuals born between April 1972 and March 1973 in Dunedin, New Zealand, who were eligible for the longitudinal study based on residence in the province at age 3 and who participated in the first follow-up assessment at age 3. The cohort represents the full range of socioeconomic status in the general population of New Zealand’s South Island and matches the NZ National Health and Nutrition Survey on adult health indicators (e.g. BMI, smoking, GP visits)[26]. The majority of cohort members are White. Assessments were carried out at ages 3, 5, 7, 9, 11, 13, 15, 18, 21, 26, 32, and most recently, 38 years, when we assessed 95% of the 1,007 study members who were still alive. At each assessment wave, Study members (including emigrants and prisoners) were brought to the Dunedin Multidisciplinary Health and Development Research Unit for a full day of interviews and examinations. These data are supplemented by searches of official records, and by questionnaires that were mailed to informants nominated by the Study members themselves. Written informed consent was obtained from all Study members. The University of Otago Ethics Committee approved each phase of the Study and the consent procedure.

### Blood collection and processing

Venous blood samples were collected from Study members at age 38 by a trained phlebotomist using standard blood collection apparatus. Blood was collected into 10ml K_2_EDTA Vacutainer tubes (BD, Franklin Lakes, NJ). After the necessary initial inversion, tubes were processed by centrifugation at 2,000g for 10 minutes (K_2_EDTA) and the plasma fraction was transferred into 2ml cryovials. All plasma was stored at -80°C until analysis.

### *T*. *gondii* IgG level measurement

Levels of IgG antibodies to *T*. *gondii* were determined using a Toxoplasma IgG Immunosimplicity EIA kit (Diamedix, FL., USA). Briefly, plasma was diluted 1 in 100 in sample diluent prior to assaying, and 100μL of diluted samples, standards (0 IU/ml, 50 IU/ml and 250IU/ml), controls (high positive, low positive and negative) and blank (sample diluent only) were placed in the reaction plate. Reactions were performed following the manufacturer’s instructions. All EIA reactions were performed in duplicate. Upon completion of the reactions, OD readings at 450nm (correcting for background at 600nm) were read using a Spectramax-384 spectrophotometer (Molecular Devices, Sunnydale, CA) and data acquired using SoftMax Pro software (Molecular Devices). Raw OD units were transformed by subtracting the mean OD value of the sample blank, and a standard curve was calculated using a point-point curve fit of the standards. The resulting equation was used to extrapolate sample OD values into IU/ml units. These values were multiplied by the initial dilution factor (1 in 100) to derive an estimate of plasma IU/ml. Duplicate values were then averaged to derive a mean IU/ml per sample. The manufacturer-recommended cut-off value for seropositivity for anti-Toxoplasma IgG using this EIA assay was 50 IU/ml, based on the WHO 3rd International Standard for Anti-Toxoplasma; Values above 50 IU/ml were designated positive for anti-Toxoplasma IgG; all other values were designated negative. The mean within-assay coefficient of variation (%CV) was 11.3%.

### Outcome measures

The measures used as outcome variables in this study are described in Table 1. Briefly, we assessed four facets of human behavior: neuropsychiatric disorder (DSM diagnosis of schizophrenia and depression), indicators of poor impulse control (non-suicidal self-injury, attempted suicide, criminal convictions, and accident claims), Big-five personality traits (Openness to Experience, Conscientiousness, Extraversion, Agreeableness, and Neuroticism), and neurocognitive performance (e.g., general intelligence, executive functions, memory, and processing speed).

**Table 1:** Brief description of neuropsychiatric, impulse control, personality and neurocognitive outcome measures used in the study.

**Outcome measure**	**Description**	
***Neuropsychiatric disorders***		
Schizophrenia	Our assessment of schizophrenia has been previously described [27]. Briefly, schizophrenia was assessed with the Diagnostic Interview Schedule (DIS) following the Diagnostic and Statistical Manual of Mental Disorders (DSM). Diagnoses required hallucinations (which are not substance use-related) in addition to at least two other positive symptoms. In addition, objective evidence of impairment resulting from psychosis was required, as reported by informants and as recorded in the Study’s life-history calendars.	
Major depression	Depression was diagnosed at age 38 using the DIS following diagnostic criteria for the DSM-IV. The reporting period was the past year.	
***Indicators of Poor Impulse Control***		
Non-suicidal self-injury	Self-reported engagement in self-injury behaviors (e.g., cutting wrists, burning self) since assessment at age 32 were ascertained at age 38 during standardized clinical interviews as well as via the Life History Calendar [28]. Behaviors only counted as non-suicidal self-injury if *not* accompanied by the intent to die.	
Attempted suicide	Self-reported suicide attempt(s) since assessment at age 32 were ascertained during standardized clinical interviews at age 38 as well as via the Life History Calendar [28]. Examples of behaviors considered attempted suicide include cutting or stabbing oneself, overdosing on pills, attempting to hang or strangle oneself, or attempting to drown. Behaviors counted as attempted suicide *only* if accompanied by self-reported intent to die.	
Criminal conviction	Linked New Zealand (NZ) government records (via the New Zealand Police) were used to determine whether Study members were convicted of any crime in adulthood, including property (e.g., theft of property of value greater than $500, receipt of stolen property, burglary, breaking and entering, shoplifting, credit car theft), court order violations (e.g., obstructing or resisting police, breaching parole, escaping prison, misleading welfare officer, failing to pay fines, failing to answer summons), drugs (e.g., possessing drug paraphernalia, supplying or procuring hard drugs or prescription medications, selling cannabis), violence (e.g., aggravated cruelty to animal, common assault, assault with intent to injure with weapon, assault of police officer, robbery, robbery aggravated with firearm, manslaughter, rape, common assault domestic) and driving convictions (including excess blood alcohol, speeding, driving without a license, causing injury, hit and run, but *not* including traffic infringements).	
Accident claims	The number of accepted accident claims per Study member from ages 21 to 38 were determined via the New Zealand Accident Compensation Corporation (ACC). The ACC provides comprehensive, no-fault personal injury coverage for all residents and visitors in New Zealand.	
***Personality at age 38***		
	At age 38, Study members nominated people "who knew them well." These informants were mailed questionnaires and asked to describe each Study member using a 25-item version of the Big Five Inventory measuring the personality traits of Extraversion, Agreeableness, Conscientiousness, Neuroticism, and Openness to Experience.	
	**Description of a Typical High Scorer**	**Description of a Typical Low Scorer**
Openness to experience	Imaginative, creative, aesthetically sensitive, quick to learn, clever, insightful, attentive and aware of feelings	Resistant to change, conventional, prefers the plain, straightforward, and routine over the complex, subtle, and abstract
Conscientiousness	Responsible, attentive, careful, persistent, orderly, planful, and future-oriented	Irresponsible, unreliable, careless, distractible, and impulsive
Extraversion	Outgoing, expressive, energetic, dominant	Quiet, lethargic, content to follow others’ lead
Agreeableness	Cooperative, considerate, empathic, generous, polite, and kind	Aggressive, rude, spiteful, stubborn, cynical, and manipulative
Neuroticism	Anxious, vulnerable to stress, guilt-prone, lacking in confidence, moody, angry, easily frustrated, and insecure in relationships	Emotionally stable, adaptable, and sturdy
***Neurocognition at age 38***		
	**Domain Tested**	**Description**
**Wechsler Adult Intelligence Scale–IV (WAIS-IV)**		The WAIS-IV [29] was administered to Study members individually according to standard protocol at age 38 years.
IQ at 38	General Intelligence	Derived from ten subtests using the method recommended in the test manual.
Verbal comprehension	Verbal Comprehension	Verbal Comprehension Index comprises the Information, Similarities, and Vocabulary subtests. The Information subtest is a test of general knowledge and reflects the ability to acquire and store knowledge in long-term memory, to access it, and to express it verbally. The Similarities subtest is a test of verbal concept formation, abstraction, and reasoning. It captures the ability to categorize and conceptualize information available in long-term memory. The Vocabulary subset is a test of language skills and includes questions about the meaning of words (e.g., What does winter mean?). It captures language processes such as the ability to acquire word meaning, recall it, and effectively express it.
Perceptual reasoning	Perceptual reasoning	Perceptual Reasoning Index comprises the Block Design, Picture Completion, and Matrix Reasoning subtests. The Block Design subtest is a test of visual-spatial organization, executive planning, and problem solving skills. The test requires putting together two, four, or nine red and white blocks in a pattern according to specific designs being displayed. The Picture Completion subtest is a test of visual discrimination and reasoning. The test involves looking at an incomplete picture of common objects or scenes and determining which part is missing. Test items are arranged in order of difficulty and have time limits. The Matrix Reasoning subtest is a test of visual-perceptual organization and reasoning ability. The test requires viewing design patterns with a missing part and selecting, from a set of five options, the part that completes the design.
Working memory	Working memory	Working Memory Index comprises the Arithmetic and Digit Span subtests. The Arithmetic subtest is a test that requires working memory processes to be applied to orally-presented verbal information. It involves numerical knowledge, short-term memory, attention, and concentration. Arithmetic problems are presented in story format (e.g., Four men can finish a job in eight hours. How many men will be needed to finish it in one-half hour?). Performance requires holding information in short-term memory, accessing long-term memory to retrieve numerical rules of mathematical operation, and using the rules to manipulate the stored data. The Digit Span subtest is a test of memory span, attention/concentration, and ability to mentally manipulate information. The test requires listening to a sequence of digits read aloud and repeating them in forward, backward, and ascending order.
Processing speed	Processing speed	Processing Speed Index comprises the Digit Symbol Coding and Symbol Search subtests. The Digit Symbol Coding subtest is a test of processing speed, psychomotor speed and coordination, and attention/concentration. Better performance also depends on incidental learning. A key that pairs symbols and numbers is presented. The test requires filling in rows containing blank squares (each with a randomly assigned number above it) using the key. The Symbol Search subtest is a test of visual processing speed, psychomotor speed and attention/ concentration. Better performance also depends on incidental learning. The test requires determining whether target symbols appear in a row of symbols.
**Trail Making Test B time**	Executive Functions	Test of scanning and tracking, divided attention, and mental flexibility [30]. The test involves drawing lines to connect consecutively numbered and lettered circles, alternating between numbers and letters. Scores represent the time, in seconds, to complete the test.
**The Cambridge Neuropsychological Test Automated Battery (CANTAB)**		The CANTAB [31] is a computerized test battery of neuropsychological functioning that uses touch-screen technology. The tests for the CANTAB have been selected based on validation in primate/rodent models and/or neuroimaging paradigms.
Rapid Visual Information Processing: A Prime	Executive Functions	Measure of sustained attention and vigilance. A white box appears in the center of the computer screen, inside which digits, from 2 to 9, appear in a pseudo-random order, at the rate of 100 digits per minute. Subjects are requested to detect target sequences of digits (for example, 2-4-6, 3-5-7, 4-6-8) and to register responses using the press pad. The signal detection measure of sensitivity to the target, regardless of response tendency (range 0.00 to 1.00; bad to good), is a measure of how good the subject is at detecting target sequences using "Probability of Hit" and "Probability of False Alarm."
Reaction Time: 5-choice Reaction time	Motor Test	Test of processing speed. The task is divided into five stages, which require increasingly complex chains of responses. In each case, the subject must react as soon as a yellow dot appears. The subject must respond by lifting their finger from the press-pad and touching the yellow dot on the screen. In some stages the dot may appear in one of five locations. The speed with which the subject releases the press pad button in response to a stimulus in any one of five randomly presented locations. Choice reaction time latency, averaged across all correct trials, is measured in milliseconds and tends toward a positive skew.
Visual Paired Associates Learning: Total errors, adjusted	Memory	Test of visual memory and new learning. Boxes are displayed on the screen and are opened in a random order. One or more of them will contain a pattern. The patterns are then displayed in the middle of the screen, one at a time, and the subject must touch the box where the pattern was originally located. If the subject makes an error, the patterns are re -presented to remind the subject of their locations. The difficulty level increases through the test. The number of patterns increases across eight stages (i.e., two 1 -pattern stage s, two 2 -pattern stages, two 3 -pattern stages, one 6 -pattern stage, one 8 -pattern stage), which challenges even very able subjects. For each stage, up to 10 trials are presented until all the patterns are located correctly. Score is the total number of errors (with an adjustment for each stage not attempted due to previous failure).
**Rey Auditory Verbal Learning Test**		Test of verbal learning and memory [32]. The test involves a five-trial presentation of a 15- word list and a one-time presentation of an interference list.
Total Recall	Memory	The total number of words (0–60) recalled over four trials (the sum of words recalled across trials 1–4).
Delayed recall	Memory	The total number of words (0–15) recalled after a 25–30 minute delay.
**Wechsler Memory Scale-III (WMS-III)**		The WMS-III [33] was administered individually according to standard protocol.
Mental Control	Executive Functions	Test of attention and tracking. It requires reciting the months of the year in backwards order, starting with December. Responses were scored according to the instructions in the WMS-III manual. Scores ranged from 1 (poor performance) to 5 (good performance) and reflect both accuracy and speed.
Paired associates: Total Recall	Memory	Test of verbal learning and memory. Eight pairs of unrelated words (e.g., truck-arrow) are read aloud and followed by a recall task (one of the words from each word pair is given, and the associated word must be recalled). Four trials of the eight word-pairs are presented. Presentation of the word-pairs is randomized across trials. The total recall score represents the total number of words (0–32) recalled across four trials.
Paired associates: Delayed Recall	Memory	The delayed recall score represents the total number of words (0–8) recalled after a 25–35 minute delay.

### Statistical analysis

The association between *T*. *gondii* infection status and the various phenotypes were tested using SPSS version 22 (IBM, New York, NY) and SAS (SAS Institute Inc., Cary, NC). We tested associations between *T*. *gondii* infection and outcomes in a regression of the form: *A* = *a* + *b1*(*T*. *gondii*) + *b2*(sex) + *e*, where *A* is one of the 27 outcome measures. The form of regression varied depending on whether the outcome under consideration represented binary or continuous data.

## Results

Plasma samples were available for 837 individuals at age 38 (423 male, 50.4%). Of these individuals, 236 (28.2%) had *T*. *gondii* IgG antibodies above 50 UI/ml, indicating a positive *T*. *gondii* infection status. This is similar to seroprevalence rates in other developed Western countries [1]. Males (32.6%) were significantly more likely to test positive for *T*. *gondii* than females (23.7%) (*χ2*(*df*) = 8.28(1), *p* < 0.01). All subsequent inferential tests included a statistical control for sex. *T*. *gondii* infection status was not related to socioeconomic status (SES); proportions of individuals testing positive for *T*. *gondii* IgG in low, medium and high SES groups were 32.2%, 29.4% and 23.4%, respectively (*χ2*(df) = 4.41(1), *p* = 0.11). We compared people with missing (N = 200) versus non-missing (N = 837) *T*. *gondii* seroprevalence data at age 38. Seroprevalence data were missing because a) for cultural reasons, Maori-ancestry cohort members’ blood was not studied (7% of cohort), b) Study members either did not consent to phlebotomy (2%), did not take part at age-38 assessment (5%), or died before age 38 (3%), or c) immunoassay data did not pass quality control (2%). Across all 27 outcome variables tested in our analysis, we found only three differences: Study members with missing *T*. *gondii* data were more likely to meet a diagnosis of schizophrenia (N = 13, 10.7%; OR (95% CI) = 4.07 (2.01–8.24), *p* < .01), a diagnosis of major depression (N = 28, 24.1%; OR (95% CI) = 1.77 (1.11–2.83), *p* = .02), and were more likely to have an adult criminal conviction (N = 54, 39.1%; OR (95% CI) = 1.60 (1.04–2.46), *p* = .03).

### 1. Is *T*. *gondii* infection status related to neuropsychiatric conditions?

We tested whether *T*. *gondii* seropositivity was associated with increased prevalence of two neuropsychiatric disorders; schizophrenia and major depression. *T*. *gondii* seropositivity was not significantly associated to either of these conditions (Table 2).

**Table 2:** The association between *T*. *gondii* infection status and the range of neuropsychiatric disorders, indicators of poor impulse control and personality.

Outcome measure	*T*. *gondii* positive	*T*. *gondii* negative	Test statistic	*p* value	Effect Size (Cohen’s *d*
	(N = 236)	(N = 601)			*(V*_*d*_*)*)
	N (%)	N (%)	Odds Ratio (95% CI)		
***Neuropsychiatric disorders***					
Schizophrenia	8 (3.4)	16 (2.7)	1.31 (0.55–3.12)	.54	.06 (0.01)
Major depression	35 (14.8)	92 (15.4)	1.01 (0.66–1.54)	.98	.00 (0.00)
***Impulsive behavior***					
Non-suicidal self-injury	10 (4.3)	19 (3.2)	1.44 (0.66–3.16)	.36	.09 (0.01)
Suicide attempt	8 (3.4)	8 (1.3)	2.63 (0.97–7.14)	.06	.23 (0.01)
Crime and accidents*					
Criminal conviction	70 (29.7)	136 (22.7)	1.19 (0.82–1.71)	.36	.04 (0.00)
Violent criminal conviction	28 (11.9)	46 (7.7)	1.36 (0.81–2.29)	.25	.07 (0.00)
Driving conviction	51 (21.6)	89 (14.9)	1.30 (0.86–1.96)	.22	.06 (0.00)
	M (SD)	M (SD)	est		
Number of Accident claims**	7.01 (6.73)	6.46 (7.59)	0.00	.99	
***Personality*, *assessed at age 38***	EMM (95% CI)	EMM (95% CI)	*F*		.00 (0.00)
Openness to experience	-0.04 (-0.17–0.09)	0.02 (-0.06–0.10)	0.62	.43	.06 (0.01)
Conscientiousness	0.01 (-0.12–0.14)	0.03 (-0.05–0.11)	0.07	.79	.02 (0.01)
Extroversion	0.00 (-0.13–0.13)	0.04 (-0.04–0.12)	0.31	.58	.04 (0.01)
Agreeableness	-0.03 (-0.16–0.09)	0.04 (-0.04–0.12)	0.82	.36	.07 (0.01)
Neuroticism	-0.01 (-0.14–0.16)	-0.01 (-0.08–0.08)	0.01	.93	.01 (0.01)

All tests are controlled for sex. N (%) represents the number and percent of individuals within each seropositivity group presenting with each phenotype. Personality scores are standardized to Mean = 0 and Standard Deviation = 1.

*Criminal conviction and accident claim data are controlled for amount of time spent in New Zealand.

**Differences tested using a zero-inflated negative binomial model;

M (SD) = mean number (standard deviation) of accepted claims. EMM = Estimated Marginal Mean. 95% CI = 95% Confidence Interval. V_d_ = Variance of Cohen’s d

### 2. Is *T*. *gondii* infection status related to poor impulse control?

We tested whether *T*. *gondii* seropositivity was associated with poor impulse control, as reflected in four phenotypes: non-suicidal self-injury, suicide attempt, criminal convictions, and traffic-related offences and accidents. *T*. *gondii* was not significantly associated with non-suicidal self-injury, but there was a suggestive link between latent infection and suicide attempt (Table 2). In regards to criminal behavior, there was no significant association between *T*. *gondii* seropositivity and criminal convictions, as reflected in official conviction records (Table 2). We further probed this non-significant association by testing whether *T*. *gondii* seropositivity was linked to certain types of criminal offenses (e.g., violence, driving) that have been previously examined in studies of *T*. *gondii* infection. *T*. *gondii* seropositivity was not significantly associated with increased risk of any type of criminal offending (Table 2).

### 3. Is *T*. *gondii* infection status related to personality differences?

In order to summarize the possible psychological differences that are associated with infection by *T*. *gondii*, we examined the personality profiles of individuals who tested positive versus negative for *T*. *gondii* antibodies. To do this, we measured the Five-Factor Model of personality. The past two decades have led to a consensus among psychologists that personality differences can be organized along five broad dimensions: Openness to Experience, Conscientiousness, Extraversion, Agreeableness, and Neuroticism [34]. Table 2 describes the mean scores across all traits for seropositive and seronegative individuals. The personality profiles of individuals who tested positive for *T*. *gondii* antibodies were indistinguishable from the personality profiles of individuals who tested negative.

### 4. Is *T*. *gondii* infection status related to poorer neurocognitive performance?

Lastly, we assessed the relationship between *T*. *gondii* seropositivity and a comprehensive range of neurocognitive functions at age 38 (Table 3). We tested whether performance on the WAIS-IV test of general intelligence at age 38 was associated with *T*. *gondii* seropositivity. There were no significant IQ differences between seropositive versus seronegative individuals. We then tested whether *T*. *gondii* seropositivity was associated with poorer performance on a range of functions encompassing verbal comprehension, perceptual reasoning, working memory, processing speed, executive functioning, motor functions and memory. *T*. *gondii* infection status was associated with poorer memory performance on the Rey Auditory Verbal Learning test. Associations between *T*. *gondii* infection status and all other tests of neurocognitive functions were not significant.

**Table 3:** The association between *T*. *gondii* infection status and neurocognitive function.

Outcome measure	Domain	*T*. *gondii* positive	*T*. *gondii* negative	Test statistic	*p* value	Effect Size (Cohen’s *d*)
		EMM (95% CI)	EMM (95% CI)	***F***		
***Neurocognition***						
**WAIS-IV**						
IQ at 38	General Intelligence	98.80 (96.83–100.77)	100.63 (99.41–101.84)	2.40	.12	.12
Verbal comprehension	Verbal Comprehension	99.12 (97.17–101.06)	100.54 (99.34–101.75)	1.50	.22	.09
Perceptual reasoning	Perceptual reasoning	98.59 (96.62–100.56)	100.32 (99.10–101.53)	2.16	.14	.11
Working memory	Working memory	98.97 (97.00–100.94)	100.64 (99.42–101.86)	2.00	.16	.11
Processing speed	Processing speed	99.43 (97.51–101.35)	100.59 (99.41–101.78)	1.03	.31	.08
**Trail Making Test B time**	Executive Functions	64.73 (62.03–67.43)	63.96 (62.30–65.62)	0.23	.63	.04
**WMS-III**						
Mental Control	Executive Functions	2.97 (2.80–3.15)	3.12 (3.01–3.23)	2.02	.16	.11
**CANTAB**						
Rapid Visual Information Processing: A Prime	Executive Functions	0.91 (0.90–0.92)	0.91 (0.91–0.92)	0.92	.34	.07
Reaction Time: 5-choice Reaction time	Motor Test	327.72 (320.96–334.48)	330.13 (325.97–334.28)	0.35	.55	.05
Visual Paired Associates Learning: Total errors, adjusted	Memory	11.85 (9.46–14.25)	12.93 (11.46–14.41)	0.57	.45	.06
**Rey Auditory Verbal Learning Test**						
Total Recall	Memory	36.76 (35.76–37.76)	37.90 (37.29–38.52)	3.64	.06	.15
Delayed recall	Memory	8.83 (8.43–9.23)	9.33 (9.08–9.58)	4.38	.04	.16
**WMS-III**						
Paired associates: Total Recall	Memory	15.63 (14.53–16.74)	16.32 (15.64–17.01)	1.07	.30	.08
Paired associates: Delayed Recall	Memory	5.09 (4.76–5.42)	5.28 (5.07–5.48)	0.89	.35	.07

All tests are controlled for sex. WAIS-IV tests are standardized to Mean = 100 and Standard Deviation = 15. EMM = Estimated Marginal Mean. 95% CI = 95% Confidence Interval.

V_*d*_ = 0.01

## Discussion

Our results suggest that a positive test for *T*. *gondii* antibodies does not result in increased susceptibility to neuropsychiatric disorders, poor impulse control or impaired neurocognitive ability. Moreover, we found no association between seropositivity and aberrant personality functions.

Some traits which have been linked previously to *T*. *gondii* seropositivity were marginally associated in our cohort. For example, we found that recently-attempted suicide was more common in seropositive individuals. This trend is consistent with previous studies, where history of suicide attempt, but not completion, has been related to both antibody titer [13] and seropositivity [15]. Similarly, our seropositive individuals performed more poorly on one test of verbal learning and memory (the Rey Auditory Verbal Learning Test), but this finding was the only significant difference among 14 tests and may have emerged by chance, since it was not evident on other tests of memory (the CANTAB visual paired associates and the Wechsler Memory Scale Paired Associates).

In contrast to previous studies, we did not observe a significant association between *T*. *gondii* seropositivity and schizophrenia. Approximately 40 reports have now been published showing links between schizophrenia and/or psychotic symptoms and infection status, leading to the suggestion that psychosis-spectrum conditions are a consequence of infection. Biological pathways have been proposed and pursued, centering on the schizophrenia-linked candidate neurotransmitter dopamine; in rodents, infection with *T*. *gondii* leads to aberrant dopamine signaling [35] and reduced expression of genes within the dopamine pathway, such as *DRD1*, *DRD5* and *MAOA* [36]. However, we found no link to schizophrenia or its associated neuropsychological deficits in our cohort.

One explanation is that our failure to detect statistically significant associations between *T*. *gondii* infection and brain and behavior impairments represents a false negative in an accumulating evidence base. False negative findings arise for a number of reasons, including low statistical power to detect associations due to small sample sizes (especially in relation to schizophrenia). In our case, the possibility of false negatives needs to be evaluated against several strengths of our study design. First, this is, to our knowledge, the most comprehensive assessment of the possible link between *T*. *gondii* infection and a variety of impairments in a single cohort. Previous positive associations have been reported across different studies, often in selected or clinical samples; for example, one study will examine the link to violence, another the link to schizophrenia, and yet another the link to self-injury, and so forth. Given that many of these impairments are correlated in the population and are characterized by comorbid presentation, it would be expected that they should be detectable through deep and comprehensive phenotyping of the same individuals drawn from a single birth cohort. Second, although our cohort is of only modest size, it is adequately powered to detect small effect sizes (*r* = 0.1). Moreover, we have previously published positive findings in this cohort using these same brain and behavior phenotypes [27, 28, 37, 38]. Third, our phenotypic assessment encompasses multiple sources including administrative records, reliable clinical interviews, well-established personality measures and standardized neurocognitive assessments, and failure to detect associations cannot be attributable to unreliable or idiosyncratic measurement practices. Fourth, we have minimized unwanted heterogeneity by studying participants who are the same chronological age. It is generally accepted that rates of infection increase as individuals age, probably due to cumulative increases in exposure opportunities [39]. By testing our hypotheses in a cohort of same-aged individuals, we were able to reduce the chance of spurious associations deriving from age-related exposure differences.

We do note two disadvantages of our study. First, *T*. *gondii* antibody status was assessed at age 38 only. We were unable to correlate the timing of acquisition of infection with subsequent changes in behavior. This is a limitation of all studies restricted to analysis of behavioral correlates post-infection, the current study included. Since infection might have influences on brain development (e.g. [40, 41]) there might developmentally-sensitive periods where effects of infection are magnified. It would be of great interest to conduct a similar investigation in a cohort of younger individuals who can be tracked longitudinally in order to determine cause and effect of the infection-behavior relationship. Disproportionate sample attrition can be a confounder in association studies, although often study design limits the ability to test and document missingness. We found evidence of selective missing data for three of our 27 outcome variables; although this observation would be expected by chance, it is still a consideration. A further consideration for all studies of *T*. *gondii* and behavioral outcomes, the present study included, is variation of both the parasite and host and the relationship to pathogenicity. There are a number of different *T*. *gondii* strains which differ in both virulence and prevalence across populations [1]. Human genetic variation, especially within the major histocompatibility complex, appears to be involved in pathogenicity of *T*. *gondii* [42]. Given these observations, some potentially interesting future directions for studies of *T*. *gondii* infection correlates are inclusion of measures of *T*. *gondii* strain and host response to infection (for example, magnitude of response to parasite antigens). This addition is necessary in order to determine whether discrepancies between reports can be ascribed to population-specific human and/or *T*. *gondii* variation.

A second explanation of our null findings is that earlier reports of links between *T*. *gondii* infection and behavioral impairments are exaggerated. Interest in the role of external organisms influencing human psychiatric and cognitive health is currently high. This is, in part, due to an increasing recognition of the role that inflammatory processes play in brain integrity [43–45], and in part due to the frustrating scientific search for biological causes with large effects in common mental disorders and processes (e.g. [46, 47]). Interest in *T*. *gondii* in particular has not only captured the imagination of researchers, but also the lay public. *T*. *gondii* is a microorganism whose source of transmission is common and relatable, as evidenced by numerous recent popular opinion pieces (e.g., “How Your Cat Is Making You Crazy”, [48]). It has been observed that the ‘hotter’ the topic and as more studies are reported and accumulate, replication becomes more difficult [49]. If we accept that the findings reported in the present article represent scenario two, then views of the link between *T*. *gondii* and aberrant behavior may need to be tempered accordingly.

In conclusion, our data do not support the hypothesis that infection by *T*. *gondii* is related to negative behavioral outcomes in a population-representative cohort of early middle-aged individuals. In the presence of conflicting reports, better research designs are needed to fully establish the extent to which *T*. *gondii* influences impairments in brain and behavior phenotypes.
